# Approach and Avoidance Behavior in Female Patients With Borderline Personality Disorder

**DOI:** 10.3389/fnbeh.2020.588874

**Published:** 2020-12-01

**Authors:** Jana Wiesenfeller, Vera Flasbeck, Elliot C. Brown, Martin Brüne

**Affiliations:** ^1^Department of Psychiatry, Psychotherapy, and Preventive Medicine, Division of Social Neuropsychiatry and Evolutionary Medicine, LWL University Hospital, Ruhr-University Bochum, Bochum, Germany; ^2^Department of Decision Neuroscience and Nutrition, German Institute of Human Nutrition, Nuthetal, Germany; ^3^Neuroscience Research Center, Corporate Member of Freie Universität Berlin, Humboldt-Universität zu Berlin, and Berlin Institute of Health, Charité-Universitätsmedizin Berlin, Berlin, Germany

**Keywords:** borderline personality disorder, emotion, approach-avoidance, cyberball, social exclusion

## Abstract

**Objectives:**

Borderline personality disorder (BPD) is portrayed by unstable relationships, fears of abandonment and heightened sensitivity to social rejection. Research has shown that these characteristics may lead to inappropriate social behavior including altered approach-avoidance behavior. However, it has remained unclear how social exclusion may affect approach-avoidance behavior in patients with BPD.

**Design:**

We assessed social approach-avoidance behavior and the impact of social exclusion in a sample of 38 patients with BPD and 40 healthy control participants.

**Methods:**

We used an explicit joystick-based approach-avoidance task (AAT) after playing a virtual ball-tossing game (Cyberball), which simulates the exclusion of the participant by two other players. In the AAT, participants were required to push or pull emotional stimuli, more specifically happy and angry facial expressions, with either direct or averted gaze direction.

**Results:**

Patients with BPD approached happy stimuli less and showed overall less differential approach-avoidance behavior toward individuals expressing positive or negative facial emotions compared to healthy participants, who showed more approach behavior for happy compared to angry facial expressions. Moreover, borderline symptom severity correlated inversely with the AAT score for happy facial expressions and positively with subjective unpleasantness during social exclusion as well as rejection sensitivity. However, social exclusion did not influence approach-avoidance tendencies.

**Conclusion:**

Patients with BPD showed altered approach-avoidance behavior, which might affect social interactions in the patient’s everyday lives and may therefore impede social interaction.

## Introduction

Borderline personality disorder (BPD) is characterized by fears of abandonment, poor emotion regulation, difficulties in impulse control, fragile self-images, and unstable relationships with significant others. Additional clinical symptoms include self-harm, abuse of alcohol or other drugs, risk-taking behavior, and stress-dependent paranoid ideation (Skodol et al., 2002). BPD frequently co-occurs with depressive symptoms and suicidal ideas (Beatson and Rao, 2013), whereby emotion dysregulation seems to be more specifically related to negative affect, emotional hypersensitivity and maladaptive coping mechanisms (Carpenter and Trull, 2013).

Difficulties in emotion regulation are known to impact patients’ tolerance of social closeness and intimacy, and often cause aversive responses such as disruption of social relationships (Skodol et al., 2002). Aside from borderline pathology, positive mood states generally foster approach, while negative affect more often causes avoidance of social interaction (Darwin, 1872; Lang et al., 1990; Chen and Bargh, 1999). Moreover, individuals tend to show a faster initiation of motor responses when in an unpleasant emotional state than in a pleasant emotional state (Beatty et al., 2016). Conversely, mood states are influenced by social stimuli such as facial expressions of emotions. Accordingly, approach-avoidance behavior is modulated by the evaluation of affective stimuli as either positive or negative. With regard to individuals with BPD, it is known that patients evaluate expressions of low mood as more severe than healthy controls, and even tend to judge neutral affective stimuli as negative. Together, this bias in appreciating other people’s emotions in more negative ways may contribute to difficulties in regulating interpersonal distance (Barnow et al., 2009; Baer et al., 2012) and hence impact patients’ approach-avoidance behavior.

To study these tendencies under laboratory conditions, researchers have developed an “approach-avoidance task” (AAT) (Roelofs et al., 2005), which requires participants to pull happy faces toward themselves and to push angry faces away using a joystick (“congruent condition”). In an incongruent condition, the opposite instruction is given, i.e., to push happy faces away and pull angry faces toward oneself. In general, arm flexion and movements toward oneself are associated with more positive feelings and arm extension and movements away from the body with more negative ones (Solarz, 1960; Cacioppo et al., 1993; Chen and Bargh, 1999; Neumann and Strack, 2000) further demonstrated that reaction times are faster for the congruent than for the incongruent condition. Based on the reaction times during incongruent and congruent conditions, effect scores can be calculated for the different emotional expressions. The effect score represents the level of approach or avoidance behavior to the stimulus material presented.

Another factor, which impacts approach-avoidance reactions is gaze direction, whereby direct gaze is readily perceived as a threat signal (Adams and Kleck, 2005). Accordingly, direct gaze, compared to averted gaze, more likely enhances social stimulus processing (George et al., 2001; Conty et al., 2007; Senju and Johnson, 2009; Mares et al., 2016).

Clinical AAT studies have shown that patients with social phobia were faster in pushing pictures showing angry and happy faces away from them compared to pictures showing neutral facial expressions, indicating stronger avoidance reactions to emotional social stimuli (Heuer et al., 2007). Similarly, individuals with spider phobia had more difficulties than controls when asked to pull pictures of spiders toward themselves (Rinck and Becker, 2007). In contrast, subjects scoring high on a psychopathy scale demonstrated a lack of avoidance of angry faces (von Borries et al., 2012). The findings in clinical depression have been mixed, with some studies showing that depressed patients had more pronounced avoidance tendencies (Seidel et al., 2010), while others were unable to demonstrate any biased responses (Radke et al., 2014). In schizophrenia, highly paranoid patients showed greater avoidance to angry expressions with averted gazes (Brown et al., 2014). A study which also includes the factor gaze reported that socially anxious individuals tended to avoid angry faces more when showing a straight gaze compared to an averted gaze (Roelofs et al., 2010).

As regards approach-avoidance behavior in patients with BPD, Kobeleva et al. (2014) used an implicit task requiring participants to pull trials with blue frames and push trials with yellow frames irrespective of the emotion (happy/angry) depicted in the frame. This AAT variant revealed no significant difference between patients with BPD and healthy controls. In contrast, Bertsch et al. (2018) observed a highly avoidant reaction to angry stimuli, while another study failed to replicate this finding (Schneider et al., 2020), However, Schneider et al. (2020) found that approach and avoidance were modulated by oxytocin administration, whereby oxytocin accelerated the avoidant response to angry faces relative to the approach condition toward happy facial expressions in patients with BPD. In another study, no impact of temperamental differences among BPD subtypes on AAT performance was found. In the BPD group, gaze was suggested to impact AAT performance, whereas no comparisons to a control group were conducted (Sleuwaegen et al., 2018).

Regarding the general impact of gaze direction in patients with BPD, Berchio et al. (2017) reported, using a 2-back gaze working memory task, that patients recognized averted gazes more quickly than straight gazes. This pattern was not found in controls, suggesting that not only facial expression but also gaze direction likely impacts emotion evaluation and possibly approach-avoidance behavior.

Another well-known clinical feature of BPD is that many patients are highly sensitive to perceived rejection (Staebler et al., 2011b). In fact, this hyper-sensitivity toward rejection can hinder the formation of a trustful and stable therapeutic relationship. Elevated rejection sensitivity is noticeable in situations suggestive of exclusion from social interactions or abandonment (Williams, 2007). Experimentally, rejection can be simulated by the Cyberball Task, a virtual ball-tossing game (Williams et al., 2000), which had previously been used in several studies, including ones in diverse clinical conditions (Eisenberger et al., 2003; Sebastian et al., 2010; Bolling et al., 2011; Maurage et al., 2012; Mooren and van Minnen, 2014; Zhang et al., 2017).

Social exclusion has negative psychological, cognitive, and physiological effects (Williams, 2007, 2009; Williams and Nida, 2011), as humans feel a need to belong to communities and social groups (Maslow, 1943). In BPD, social exclusion is known to produce more negative affect in patients compared to controls, and changes in plasma oxytocin in opposite directions (i.e., decrease in oxytocin upon social exclusion in BPD; Jobst et al., 2014). However, to the best of our knowledge, no study has examined approach-avoidance behavior in BPD patients following social exclusion.

Accordingly, given the clinical relevance of difficulties in the regulation of interpersonal proximity in individuals with BPD, the present study sought to investigate approach-avoidance behavior in response to emotional stimuli in patients with BPD compared to healthy control participants. Moreover, we were specifically interested in the question whether social exclusion would distinctively impact on patients’ approach and avoidance behavior. We hypothesized that patients with BPD would show more pronounced avoidance tendencies regarding negative emotions, and that social exclusion prior to the AAT would inflate this aversive response. Furthermore, we expected that patients with BPD would experience social exclusion as more unpleasant than control participants, and that patients would perceive straight gaze as more threatening than averted gaze.

## Materials and Methods

### Participants

Thirty-eight patients diagnosed with BPD, according to DSM-IV criteria (confirmed by a SKID interview, German version (Wittchen et al., 1997) were recruited from psychiatric in-patient and out-patient services of the LWL University Hospital Bochum. For comparison, forty healthy controls (HC) were recruited via advertisement. The presence of any psychiatric condition was ruled out using the Mini-DIPS (Margraf, 1994). All participants were female, aged between 18 and 56 years. The mean age of patients was 27.68 (SD = 8.17) years and the mean age of healthy controls was 26.1 (SD = 9.59) years. There was no significant difference in age between the two groups [*t*(76) = −0.95, *p* = 0.35]. Furthermore, verbal intelligence was measured using the Mehrfachwahl-Wortschatz Test [MWT-A; (Lehrl, 1991]. Participants with IQ-scores below 90 were excluded from the study. Patients with other psychiatric diagnoses such as psychotic or bipolar disorders, attention deficit hyperactivity disorder, as well as patients with addiction to alcohol or other illegal psychotropic substances were also excluded from the study. Details about medication taken by the patients and about comorbid disorders are summarized in Table 1. All participants had normal or corrected-to-normal vision. They all gave their informed consent in writing. The study was approved by the Ethics Committee of Ruhr-University Bochum (Registration number 18-6367) and in accordance with the Declaration of Helsinki.

**TABLE 1:** Frequency of Medication and comorbid disorders in BPD participants in absolute (*N*) and relative (%) quantities.

	***N***	**%**
**Medication**		
No medication	14	36
Antidepressants	14	36
Antidepressants and antipsychotic drugs	11	28
**Comorbid disorders**		
Depressive episode	18	46
Posttraumatic stress disorder	11	28
Phobic disorder	3	8
Eating disorder	3	8
Cannabis misuse	2	5
Alcohol misuse	4	10
Other substances misuse	6	15
Obsessive-compulsive disorder	1	3

### Questionnaires

#### BSL-23

Symptom severity was assessed using the BSL-23 (Bohus et al., 2009). A general BSL-23 score and a behavioral score, assessing BPD associated behavior in the last 7 days were obtained.

#### RSQ

Sensitivity to rejection was examined using the German version of the Rejection Sensitivity Questionnaire (Downey and Feldman, 1996; Staebler et al., 2011a).

### Tasks

#### Facial Emotion Recognition Task

Prior to the AAT (see below) participants performed a facial emotion recognition task (FERT) to control for deficits in emotion recognition, which could potentially influence AAT performance. The task was based on the pictures of facial affect (PFA) test, a categorization task, which had been used in previous studies (Frommann et al., 2013; Luckhaus et al., 2013). Stimuli were chosen from the NimStim Set of Facial Expressions, a database with over 600 full color emotional stimuli (Tottenham et al., 2009), which has widely been used for research purposes (Houston et al., 2018; Mowle et al., 2019).

**FIGURE 1:**
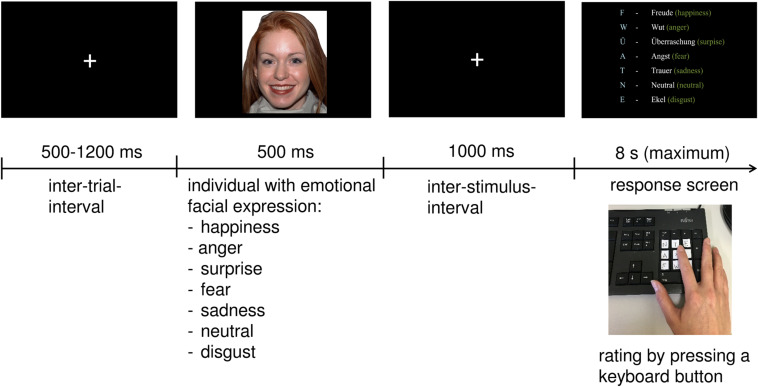
Description of the Facial Emotion Recognition task.

After the presentation of a fixation cross for 500–1,200 ms, 70 facial expressions (35 males and 35 females) were presented on a screen for 500 ms. Within the 70 trials, seven different emotions were displayed in random order: anger, disgust, sadness, happiness, fear, surprise, or simply showed a neutral facial expression. After another fixation cross occurring for 1,000 ms, participants had to answer as quickly and as accurately as possible by selecting the corresponding key on the keyboard. The response screen was presented for a maximum of 8 s. When the response was given, the next trial started. The keys were marked with a sticker with the first letter of each emotion (see Figure 1).

#### Cyberball Game

Participants were divided into two groups, with half of them playing the Cyberball Game prior to the AAT, while the other half was watching a short and relaxing documentary as control condition.

Participants were told they would be playing a ball-tossing game with two other players over the internet who were seated in other rooms in the hospital. For this purpose, a photograph of each participant was taken, and they were explained that it would be their profile picture used during the game. The task was to throw the ball to another player by clicking on his/her profile picture. Unbeknownst to the participant, the other players were computer-generated. To create authenticity, intervals for the virtual players to throw the ball differed between 1 and 5 s, so that it appeared to be the time the other needed to decide to whom to throw the ball. In total, the task lasted 3 min with equal numbers of ball contacts between all three players during the first 15 trials. From the 15th to the 30th trial the participant did not receive any more tosses.

After finishing the task, participants were asked to evaluate how unpleasant it had been for them to be excluded and how angry it made them (1–10) in order to measure the subjective impact of social exclusion.

The documentary watched by the other half of participants was about yoga (Indian Diplomacy, 2015), and was shown for 3 min. Both, the Cyberball Game and the documentary, were performed directly before the AAT.

#### AAT

The AAT required participants to use a joystick (Logitech Attack 3). Individuals were seated in front of a 21.5″ screen with the joystick being placed on the table in front of the screen. Participants were instructed to start the task by pushing the start button on the joystick. The images used were black-and-white photographs of facial expressions taken from Ekman and Friesen’s PFA (Ekman and Friesen, 1976) and the Karolinska Institute database (Lundqvist et al., 1998). These stimuli were also used in previous AAT studies (Roelofs et al., 2010; Brown et al., 2014). Over a period of 12 min, photographs of four women and four men were shown, with half of the pictures depicting happy faces and half of them displaying angry facial expressions. Moreover, half of them had a direct gaze and half of them an averted one.

The AAT was given in two versions: the congruent and the incongruent condition with half of the participants starting with the congruent condition and proceeding with the incongruent one, and the other half vice versa. They were pseudo-randomly assigned to the groups, with every second participant starting with the congruent condition and every second one with the incongruent condition.

For the congruent condition, participants were told to pull happy faces toward themselves and to push angry faces away from them. For the incongruent condition they were told to push happy faces away from them and pull angry faces toward them. For both conditions, the instruction was to respond as quickly and accurately as possible.

**FIGURE 2:**
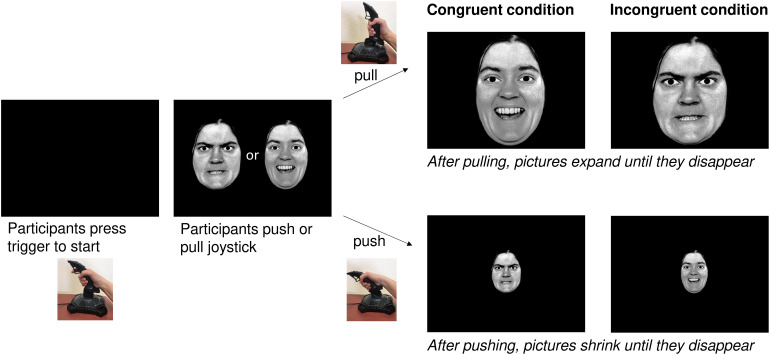
Description of the AAT.

Once the movement of the joystick had started, the image increased in size when the joystick was pulled toward oneself and decreased in size when the joystick was pushed away from oneself. Reaction times (RTs) were measured starting once the joystick was moved from its resting position (see Figure 2).

As in previous studies all incorrect trials (Brown et al., 2014) as well as all trials with a RT < 200 ms and >1,000 ms were excluded from the analyses (response accuracy is shown in Table 2). For each emotion and gaze direction, AAT scores were determined, resulting in four effect scores: happy-straight gaze, happy-averted gaze, angry-straight gaze, angry-averted gaze. They were calculated by subtracting push-pull RTs (Roelofs et al., 2010; Brown et al., 2014). A higher score can be interpreted as a greater approach tendency, whereas a lower score represents a greater avoidance tendency (Roelofs et al., 2005). AAT reaction times are presented in Table 3.

**TABLE 2:** Mean percentage and coefficient of variation of accurate AAT trials.

			**BPD [Mean (CV)]**	**HC [Mean (CV)]**
Congruent	Happy	Straight	70.38% (28.13%)	86.62% (12.15%)
		Averted	66.44% (37.54%)	85.96% (9.26%)
	Angry	Straight	72.64% (24.10%)	83.44% (12.88%)
		Averted	73.31% (23.47%)	86.51% (13.91%)
Incongruent	Happy	Straight	68.47% (27.38%)	79.39% (15.88%)
		Averted	66.67% (26.68%)	80.60% (16.36%)
	Angry	Straight	72.52% (22.61%)	81.25% (12.95%)
		Averted	69.48% (25.56%)	82.79% (14.01%)

**TABLE 3:** Means and standard deviations of AAT reaction times in ms in patients with BPD and HC.

**Condition**	**Emotion**	**Gaze**	**BPD [Mean (SD)]**	**HC [Mean (SD)]**
Congruent	Happy	Straight	651.43 (54.16)	615.25 (52.00)
		Averted	665.16 (59.76)	628.37 (49.22)
	Angry	Straight	666.94 (40.59)	643.82 (55.40)
		Averted	669.33 (53.33)	640.18 (50.30)
Incongruent	Happy	Straight	668.27 (51.55)	657.68 (51.06)
		Averted	679.06 (51.52)	658.11 (51.61)
	Angry	Straight	661.13 (44.07)	641.13 (52.16)
		Averted	659.63 (42.78)	647.69 (48.64)

### Procedure

Firstly, the FERT was performed, followed by the documentary or Cyberball task and the AAT. Questionnaires were filled out after the experimental procedure.

### Data Analysis

All statistical analyses were carried out using IBM SPSS Statistics for Windows, version 25 (IBM Corp., Armonk, NY, United States). A significance level of *p* < 0.05 was chosen for all tests. All values from ANOVAs were Greenhouse-Geisser corrected. Questionnaires were analyzed using independent *t*-tests or Mann–Whitney *U* tests (BSL-23). Regarding the FERT, the data were not normally distributed since some emotions were recognized perfectly by almost all participants, leading to a ceiling effect. Thus, a Kruskal-Wallis-Test was carried out with Group (BPD, HC) as grouping variable and the Emotions (joy, anger, sadness, fear, surprise, disgust, and neutral expression) as test variables.

For the analysis of the AAT, we performed a mixed-model ANOVA with the factors “Emotion” (happy, angry) and “Gaze” (straight, averted) and the between subject factors “Group” (BPD, HC) and “Condition” (Cyberball, Documentary). Thus, the whole sample was divided into BPD and HC groups and further split into Cyberball and Documentary groups. Moreover, in order to control whether observed differences in the recognition of happy facial expressions (FERT) had an influence on the outcome of the AAT, the emotion “happy” (FERT) was introduced as a covariate in the mixed-model ANOVA. For further *post hoc* comparisons, dependent and independent *t*-tests were used. Additionally, Pearson correlation coefficients were calculated between the evaluation of the Cyberball game, BSL-23 scores, and the AAT happy effect scores “happy” and “angry” (both include straight and averted gazes). Finally, as almost half of the group were diagnosed with a depressive episode, an additional independent *t*-test was conducted checking for differences in effect scores between patients with vs. patients without comorbid depression.

## Results

### RSQ, BSL-23, and Cyberball Evaluation

A Mann-Whitney test indicated that the BSL-23 score was higher in BPD patients (Median = 59.0) than healthy controls (Median = 20.5), *U* ≤0.001, *p* ≤0.001. The behavioral score was also higher in patients (Median = 57.15) compared to healthy controls (Median = 22.21), *U* = 68.5, *p* ≤0.001. Furthermore, patients with BPD (Median = 58.55) were significantly more sensitive to rejection (RSQ) compared to healthy control participants (Median = 20.9), *U* = 16.5, *p* ≤0.001. Concerning the evaluation of the Cyberball game, patients were asked how unpleasant it had been and how angry it had made them to have been excluded. The results showed that patients felt more unpleasant and angrier than healthy controls (unpleasant/angry: *M* = 6.23, SD = 3 vs. *M* = 2.82, and SD = 2.4) [*t*(36) = -3.8, *p* = 0.001] In addition, a positive correlation between BSL scores, rejection sensitivity and Cyberball evaluation emerged; that is, higher BSL values correlated with more rejection sensitivity as well as a more negative evaluation of the Cyberball game (see Table 4).

**TABLE 4:** Correlations between AAT happy effect scores, BSL-23, Cyberball evaluation and rejection sensitivity questionnaire (RSQ) score.

	**1**	**2**	**3**	**4**	**5**
1. AAT angry	–	−	0.13	0.1	0.146
2. AAT happy		−	−0.25*	−0.02	−0.173
3. BSL-23			−	0.46**	0.832**
4. Cyberball unpleasantness				−	0.504**
5. RSQ					−

Results reported are correlation coefficients (*r*) with significant correlations printed in bold and *indicating correlations with *p* < 0.05 and **representing *p* ≤ 0.001.

### FERT

Kruskal–Wallis comparisons revealed significant differences between healthy controls and BPD patients for happy and neutral trials [happy: *H*(1) = 4.77, *p* = 0.029; neutral: *H*(1) = 4.5, *p* = 0.035]. There was no significant difference regarding the other emotions. This indicated that patients with BPD made more errors in recognizing neutral and happy facial expressions, compared to healthy controls (happy: Median = 34.67 vs. Median = 44.09; neutral: Median = 34.75 vs. Median = 44.01). A closer look at happy trials revealed that the observed difference between groups for happy stimuli was significant for the recognition of male facial expressions [*H*(1) = 3.91, *p* = 0.048], but not female facial expressions [*H*(1) = 0.89, *p* = 0.35].

### AAT

**FIGURE 3:**
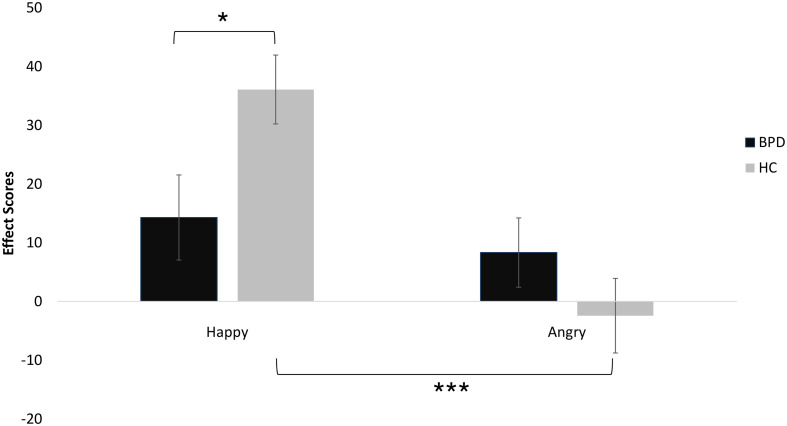
AAT performance of patients with BPD and unaffected controls (error bars denote one standard error around the mean). Significant differences are marked with *p < 0.05 and ***p < 0.001.

The mixed-model ANOVA with “Emotion” (happy, angry) and “Gaze” (straight, averted) as within-subject factors, the between subject factors “Group” (BPD, HC) and “Condition” (Cyberball, Documentary), and “Happy” (FERT) as covariate displayed an Emotion × Group interaction [*F*(1,76) = 5.55, *p* = 0.021, partial η^2^ = 0.068] (see Figure 3). There were no main effects for “Emotion” or “Gaze”, and there were no interaction effects for “Emotion” × “Condition”, “Condition” × “Gaze” or “Gaze” × “Group”. Concerning the “Emotion” × “Group” interaction, *post hoc* analyses revealed a significant difference between happy and angry affect scores [*t*(77) = 3.09, *p* = 0.003], whereby the difference between the two types of facial emotions was only significant in controls [*t*(39) = 3.89, *p* ≤0.001] but not in BPD patients [*t*(37) = 0.58, *p* = 0.57].

Furthermore, happy effect scores differed significantly between groups [*t*(76) = 2.33, *p* = 0.022], whereas there was no significant difference for angry effect scores [*t*(76) = -1.23, *p* = 0.223; see also Figure 3]. Importantly, effect scores did not significantly differ between patients with and patients without comorbid depression [happy: *t*(37) = 0.68, *p* = 0.5; angry: *t*(37) = 0.52, *p* = 0.6].

Regarding the associations between questionnaires and AAT effect scores, a significant inverse correlation was found between the score for happy facial expression and the BSL-23 score (*r* = -0.25, *p* < 0.05; see Table 2).

## Discussion

This study sought to investigate social approach-avoidance behavior in response to social exclusion in patients with BPD. During the AAT task, patients with BPD approached happy stimuli more slowly and presented less pronounced approach behavior in general, whereas no difference between BPD and HC occurred regarding angry stimuli. Contrary to predictions, gaze direction (straight/averted) did not have an impact on approach/avoidance behavior either. Regarding social exclusion, patients felt angrier and more unpleasant after being excluded in the Cyberball game compared to the healthy control group. However, this finding did not have a differential effect on approach-avoidance behavior.

Interestingly, the severity of BPD symptoms correlated negatively with approach behavior toward happy facial expressions, indicating that high BPD symptoms were related to low approach behavior to happy facial expressions. This finding is in contrast with a study reporting no difference in approach behavior between BPD patients and controls (Kobeleva et al., 2014). Our own and Kobeleva et al. (2014) study are, however, not directly comparable, because Kobeleva et al. (2014) utilized an implicit approach, whereby participants responded to a distracter stimulus (color), rather than facial emotion directly. When explicitly asked to rate the depicted person’s approachability, patients with BPD responded in more negative ways than controls, and this negative attitude corresponded to the severity of BPD symptoms (Kobeleva et al., 2014).

Possible explanations for the reluctance to approach individuals with happy facial expressions could reside in the fact that patients with BPD often experience others as untrustworthy (King-Casas et al., 2008; Unoka et al., 2009; Miano et al., 2013) or unapproachable (Nicol et al., 2013). Alternatively, it may relate to a general negative evaluative style (Baer et al., 2012) or the fact that positive emotions are experienced less in BPD (Lenzenweger et al., 2004; Reed and Zanarini, 2011). Together these possible explanations are not mutually exclusive and difficult to disentangle experimentally.

No differential effect occurred for angry faces, which is in line with previous findings (Kobeleva et al., 2014; Schneider et al., 2020), although one study found less pronounced approach-avoidance behavior in anger-prone women with BPD (Bertsch et al., 2018). Our results are coherent with the idea that psychophysiological reactions to negative effects appear to be intact in BPD (Herpertz et al., 1999; Kuo and Linehan, 2009; Dukalski et al., 2017).

Another interesting finding was that BPD patients distinguished less between positive and negative emotions (as shown in their indiscriminate approach-avoidance response to happy versus angry faces), whereas healthy controls clearly showed differential responses especially concerning the approach response to happy facial expressions. A speculative explanation could be that disorganized attachment in BPD (Agrawal et al., 2004; Gunderson, 2007) may cause insecurity concerning approach-avoidance behavior, particularly in individuals who experienced their primary caregivers as a source of both care and threat (Main and Solomon, 1986). Consistent with our results, less distinctive approach-avoidance tendencies have been seen in patients with depression (Radke et al., 2014), while another study reported more pronounced avoidance tendencies in female patients with depression (Seidel et al., 2010). As comorbid depression was high in our sample, we cannot rule out that depressive mood played a role in AAT performance.

Surprisingly, patients with BPD did not respond differently to straight vs. averted gazes. This is somewhat counter-intuitive, as patients with BPD seem to be sensitive toward gaze direction (Berchio et al., 2017), as well as sensitive toward perceived threat. A possible explanation could reside in the setup of our task, because the pupils were quite difficult to discern from the surrounding iris in the B/W images of facial affect, such that the determination of gaze direction was solely based on the visibility of the sclera.

A second goal of the present study was to investigate the effect of social exclusion on approach-avoidance behavior. As expected, patients with BPD felt angrier and more unpleasant about being excluded in the Cyberball game than the healthy control group. A positive correlation was found between BPD symptom severity, rejection sensitivity and subjective unpleasantness of the Cyberball game. These findings are compatible with previous Cyberball studies in patients with BPD (Renneberg et al., 2012; Domsalla et al., 2014; Euler et al., 2018), corroborating the finding that rejection hypersensitivity is a core feature of BPD (Staebler et al., 2011a,b; Bungert et al., 2015). Contrary to expectations, however, performance in the AAT was unaffected by prior social exclusion. That is, patients who felt a high negative impact of the Cyberball game did not differ in performance in the AAT from patients with lower scores. Similarly, in healthy controls, after being excluded in the Cyberball game, no change in interpersonal approach-avoidance behavior in the AAT was found (Hess et al., 2018).

Approach-avoidance behavior after social exclusion may be influenced by two different behavioral goals: one is the desire to recuperate inclusion (DeWall and Richman, 2011), or the need to protect oneself from ongoing rejection (DeWall and Bushman, 2011). Our prediction was that the latter were the case in the BPD group, which turned out to be wrong. Even though speculative at this point, it could be the case that approach-avoidance behavior is a more reflective process (Phaf et al., 2014), such that possible changes in approach-avoidance behavior after social exclusion may not be discernible in the AAT, which taps into more immediate and stimulus-based reactions.

The present study has several limitations. First, only female participants were included such that the findings cannot be generalized for both sexes. Since it is well known that males and females process facial emotions differently, a replication including male participants is warranted (Kret and de Gelder, 2012). Additionally, it should be taken into account that the menstrual cycle may affect BPD symptom expression (Peters and Eisenlohr-Moul, 2019), a factor that was not considered in this study. Second, as almost half of the patients were diagnosed with comorbid depression, we cannot rule out confounding effects of depressed mood. However, the performance of the clinical sample in the FERT was relatively typical for individuals with BPD, and results remained stable, even if performance in the FERT were statistically controlled for. Another aspect that could not be controlled was the potential impact of psychotropic medication, which is relevant because antidepressants tend to influence emotion processing (Kerestes et al., 2009; Brühl et al., 2011). Moreover, results regarding gaze direction in the AAT should be interpreted cautiously, as it remains unclear as to what extent approach-avoidance behavior is triggered by gaze direction or facial expression. Future research should consider eye-tracking and the use of color images. Finally, even though the Cyberball game is a common research tool for mimicking social exclusion, its “real-life” validity is limited.

## Conclusion

Patients with BPD displayed attenuated approach behavior toward happy facial stimuli and seem to discriminate less between positively and negatively valenced PFA. Moreover, they seemed to respond in more sensitive ways upon social exclusion, although this did not impact approach or avoidance. In future studies it may be useful to distinguish subgroups based on comorbidity profile and perhaps according to more specific personality traits such as externalizing versus internalizing behavior. Moreover, the investigation of effects of the menstrual cycle on social approach and avoidance behavior should be taken into account in future studies.

## Data Availability Statement

The raw data supporting the conclusions of this article will be made available by the authors, without undue reservation.

## Ethics Statement

The studies involving human participants were reviewed and approved by Ethics Committee of the Medical Faculty of the Ruhr University Bochum. The patients/participants provided their written informed consent to participate in this study.

## Author Contributions

EB, MB, and VF designed the experiment and revised the first version. JW carried out data collection and wrote the first draft of the manuscript. JW, VF, and MB performed the data analysis. All authors endorsed the final version.

## Conflict of Interest

The authors declare that the research was conducted in the absence of any commercial or financial relationships that could be construed as a potential conflict of interest.
